# Circulating miR-221/222 and Serum IL-23 in Treatment-Naïve Multiple Sclerosis: A Case–Control Study

**DOI:** 10.3390/medicina62081468

**Published:** 2026-07-29

**Authors:** Ummu Serpil Sarı, Nermin Tepe, Ayla Solmaz Avcıkurt, Saliha Uysal, Hilmi Bolat, Figen Eşmeli

**Affiliations:** 1 Department of Neurology, Faculty of Medicine, Balikesir University, 10145 Balikesir, Turkey; tepenermin@gmail.com; 2 Department of Medical Genetics, Faculty of Medicine, Balikesir University, 10145 Balikesir, Turkey; aylaavcikurt@gmail.com (A.S.A.); hilmi_bolat@hotmail.com (H.B.); 3 Department of Medical Biochemistry, Faculty of Medicine, Balikesir University, 10145 Balikesir, Turkey; drsaliha87@gmail.com; 4 Department of Neurology, Faculty of Medicine, Izmir University of Economics, 35330 Izmir, Turkey; fesmeli@hotmail.com

**Keywords:** multiple sclerosis, miR-221, miR-222, IL-23, microRNA expression, ΔΔCt, RT-qPCR, exploratory biomarker study

## Abstract

*Background and Objectives*: Circulating microRNAs (miRNAs) are emerging as accessible biomarkers for multiple sclerosis (MS). In experimental models, miR-221 and miR-222 have been linked to immune and Th17-related pathways, while interleukin-23 (IL-23) is a cytokine driving autoimmune inflammation. This observational study evaluated the expression of circulating miR-221/222 and serum IL-23 concentrations in treatment-naive adult patients with MS. *Materials and Methods*: This prospective, cross-sectional case–control study included 43 untreated adult patients with MS (36 with relapsing–remitting MS and 7 with primary progressive MS) and 37 healthy controls. Demographic features, Expanded Disability Status Scale (EDSS) scores, magnetic resonance imaging involvement, initial symptoms, serum IL-23 concentration, and miR-221/222 expression were recorded. Total RNA, including small RNAs, was isolated; complementary DNA was synthesized by reverse transcription from RNA templates; and reverse transcription–quantitative real-time PCR (RT-qPCR) was performed using RNU6-2 as the endogenous small RNA reference. Individual ΔΔCt values were specified as the primary inferential scale, while 2^−ΔΔCt^ fold-change was retained only for descriptive reporting. *Results*: The relative expression (2^−ΔΔCt^) of miR-221 and miR-222 was higher in patients than in controls. Within the patient group, miR-221 and miR-222 relative expression did not differ by age, sex, EDSS, time since MS diagnosis, MRI involvement area, or initial symptoms. No statistically significant difference in IL-23 levels was observed between the patient and control groups. *Conclusions*: The presence of unaltered IL-23 levels alongside significantly elevated miR-221 and miR-222 expressions in treatment-naive MS patients during the remission phase suggests their potential utility as biomarkers for immune regulation. Given the cross-sectional design of this study, no causal or regulatory relationship between miR-221/222 and IL-23 can be inferred.

## 1. Introduction

Multiple sclerosis (MS) is a chronic, autoimmune, demyelinating, and neurodegenerative disease of the central nervous system (CNS) [1]. It predominantly affects women, with a peak onset typically between 20 and 40 years of age. Although the precise etiology of MS remains elusive, genetic predisposition interacting with environmental and epigenetic factors plays a pivotal role in disease development [2]. The pathogenesis of MS involves diverse interconnected mechanisms, including peripheral inflammatory immune activation, blood–brain barrier dysfunction, and the abnormal activation of glial cells and T-lymphocytes [3]. Clinical courses of MS are classified into distinct phenotypes, including relapsing–remitting MS (RRMS), primary progressive MS (PPMS), and secondary progressive MS (SPMS), with RRMS being the most prevalent subtype [4].

miRNAs are short, single-stranded, non-coding RNAs typically 18 to 25 nucleotides in length that regulate gene expression post-transcriptionally through complementary base pairing; they play essential roles in modulating cell signaling pathways, including those regulating oncogenes or tumor suppressors [5]. To date, approximately 2000 miRNAs have been identified in the human genome. Given their central regulatory functions within both the immune and central nervous systems, miRNAs have emerged as an important area of investigation in autoimmune and neurological disorders, particularly MS [6,7].

miRNAs are crucial for neurogenesis, oligodendrocyte differentiation, and myelin formation and exert their functions post-transcriptionally by binding to complementary sequences within the 3′ or 5′ untranslated regions (UTRs) of target messenger RNAs (mRNAs). For instance, miR-223 has been shown to facilitate the efficient activation and phagocytic clearance of myelin debris by microglia, whereas miR-204, miR-138, and miR-219 promote the maturation and differentiation of oligodendrocytes. Additionally, other miRNAs, including miR-145 and miR-338, are actively involved in the synthesis and assembly of myelin-specific proteins [8,9,10,11].

The miR-221/222 cluster is widely implicated in oncogenesis as well as the regulation of inflammatory and immune cascades [12]. Notably, while the upregulation of miR-221/222 in endothelial cells suppresses cell growth, proliferation, and angiogenesis. Paradoxically, its activity is required for cancer cell proliferation via accelerating cell cycle progression through the targeting of the cell-cycle inhibitor p27 [13]. Furthermore, circulating miRNA profiles vary in neuroinflammatory states; a comparative study involving 58 MS patients (54 RRMS and 4 SPMS) and 20 healthy controls demonstrated a significant upregulation of miR-320a, miR-125a-5p, miR-652-3p, miR-185-5p, miR-942-5p, and miR-25-3p [11].

Beyond central nervous system disorders, the systemic relevance of miR-221 has been documented in other autoimmune conditions. In patients with rheumatoid arthritis, miR-221 expression is significantly elevated in both serum and synovial tissues; conversely, its downregulation inhibits the secretion of proinflammatory cytokines and chemokines, suppresses the migration and invasion of fibroblast-like synoviocytes, and induces apoptosis [14]. In contrast to its inflammatory role, the in vitro upregulation of miR-221/222 in Schwann cells has been shown to promote the expression of nerve growth factor (NGF) and myelin basic protein (MBP), highlighting its complex, context-dependent regulatory capacity [15].

IL-23 is another factor that appears to be a critically important cytokine in MS pathogenesis. In an experimental autoimmune encephalomyelitis (EAE) model, astrocyte-specific expression of IL-23 has been shown to exacerbate the disease phenotype, leading to B-cell accumulation and an amplified inflammatory response [16]. Under physiological and pathological conditions, IL-23 is secreted by activated macrophages and dendritic cells in the lungs, intestinal mucosa, and skin, subsequently driving the differentiation and maintenance of T helper 17 (Th17) cells. Furthermore, it modulates antibody production, enhances T-cell proliferation, and promotes the activation of natural killer (NK) cells [17]. The clinical significance of IL-23 across various autoimmune inflammatory conditions—including inflammatory bowel disease, psoriasis, and rheumatological disorders, alongside MS—has been reported [18]. Upon antigen-specific stimulation, IL-23 contributes to the expansion of activated CD4+ T cells that produce IL-17, IL-6, and tumor necrosis factor (TNF), thereby playing a pivotal role in autoimmune inflammation [19]. Mechanistically, IL-23 is a proinflammatory cytokine that exerts its biological actions by binding to its functional receptor complex composed of IL-23R and IL-12Rβ1 [17,20].

As miRNAs are critical regulators of immune responses, recent evidence has highlighted the role of the miR-221/222 cluster in modulating intestinal Th17 cell dynamics. Proinflammatory cytokines have been shown to upregulate miR-221 and miR-222 expression, whereas the regulatory cytokine transforming growth factor-beta (TGF-β) represses this activity. Structurally, miR-221 and miR-222 directly target Maf and *IL23r*; consequently, the genetic loss of miR-221 and miR-222 shifts the transcriptomic landscape of intestinal Th17 cells toward a more pronounced proinflammatory signature [21].

Studies have highlighted the future importance of miR-221/222 as a promising biomarker and molecular target for cancer and inflammatory diseases. In our research, we sought to examine the contribution of miR-221/222 and the association between IL-23 levels and miR-221/222 in newly diagnosed RRMS and progressive MS patients without treatment. We therefore examined whether circulating miR-221/222 relative expression in untreated MS patients is associated with clinical disability (EDSS), MRI lesion burden, and serum IL-23, and whether these miRNAs might serve as candidate biomarkers; any regulatory role in these pathways is hypothesized from prior experimental work rather than demonstrated by the present study.

## 2. Materials and Methods

### 2.1. Study Design and Participants

This was a prospective, cross-sectional case–control study of 43 patients aged ≥ 18 years who were followed up at the Neurology Outpatient Clinic of Balikesir University Hospital. Patients had newly diagnosed, treatment-naïve RRMS or primary progressive MS according to the 2017 McDonald Criteria [22]. A patient was defined as treatment-naïve if he or she had received no prior disease-modifying therapy at any time before sampling. Thirty-seven healthy volunteers who had no illness and were not receiving any medication or immunomodulatory treatment before sample collection were included as the control group. Detailed neurological examinations, EDSS (Expanded Disability Status Scale) scores, attack frequencies, attack types (visual, motor, sensory, and spinal attacks), and neuroimaging (cranial, spinal, and orbital MRI [Magnetic Resonance Imaging]) were documented. Individuals who were obese (BMI [Body Mass Index] > 25) or who had chronic renal failure, liver dysfunction, inflammatory, autoimmune, or rheumatologic disease, or an oncologic diagnosis, or who were using immunosuppressive or immunomodulatory drugs, were excluded.

The study was approved by the Ethics Committee of Balikesir University (approval number 2022/119), and written informed consent was obtained from all participants. This study was financially supported by the Balikesir University Scientific Research Project Unit (2021/131).

### 2.2. Blood Sampling and IL-23 Measurement

Approximately 5–10 mL of venous blood was drawn from both groups into a gel biochemistry tube and centrifuged at 1800× *g* for 10 min. The serum samples were stored in Eppendorf tubes at −80 °C. Human IL-23 ELISA kit (Shanghai Sunred Biological Technology Co., Ltd., Shanghai, China; Cat. No: SRB-T-83268) analyses were performed on the stored serum samples using the Enzyme-Linked Immunosorbent Assay (ELISA) method. The measurement range for IL-23 was 0.6–180 pg/mL, and the intra-assay CVs were below 10%.

### 2.3. Gene Expression

Peripheral venous blood samples were obtained from 43 patients with multiple sclerosis and 37 healthy controls. Total RNA, including small RNAs, was extracted using the SanPrep Column MicroRNA Mini-Prep Kit (Bio Basic Inc., Markham, ON, Canada; Cat no.: SK8811) according to the manufacturer’s protocol. Complementary DNA (cDNA) was synthesized by reverse transcription of the extracted total RNA (including the small-RNA fraction) using the miRNA All-In-One cDNA Synthesis Kit (Applied Biological Materials Inc., Richmond, BC, Canada; Cat no.: G898).

RT-qPCR was performed using BlasTaq 2X qPCR MasterMix (Applied Biological Materials Inc., Richmond, BC, Canada; Cat no.: G891, G892) on a Rotor-Gene Q system (Qiagen, Düsseldorf, Germany). All reactions were performed in triplicate. Specific primers were used for hsa-miR-221, hsa-miR-222, and RNU6-2, which served as endogenous small RNA controls (*MIR221 Human qPCR Primer Pair* Forward = ACCTGGCATACAATGTAG Reverse = GAACATGTCTGCGTATCTC Locus ID: 407006 *MIR222 Human qPCR Primer Pair* Forward = CTCAGTAGCCAGTGTAG Reverse = GAACATGTCTGCGTATCTC Locus ID: 407007). RNU6-2 was used as the endogenous reference because it is processed and co-extracted similarly to mature miRNAs and is stably and abundantly expressed; a protein-coding mRNA reference (e.g., *ACTB*) was deliberately not used, as mRNAs differ from miRNAs in biogenesis and extraction efficiency and would introduce systematic normalization bias.

Gene expression levels were determined based on the threshold cycle (Ct) values. ΔCt values were calculated as Ct_target − Ct_RNU6-2 for each sample. ΔΔCt was calculated as ΔCt_target sample − ΔCt_calibrator sample. Relative expression levels were determined using the 2^−ΔΔCt^ method, whereby the calibrator group was assigned a value of 1, as ΔΔCt = 0, and therefore, 2^0^ = 1 [23,24,25,26].

Amplification efficiencies for miR-221, miR-222, and RNU6-2 were evaluated using a five-point 10-fold serial dilution standard curve. The slope values were −3.41 for miR-221 (97% efficiency), −3.36 for miR-222 (98% efficiency), and −3.34 for RNU6-2 (99% efficiency), with R^2^ values greater than 0.99. The efficiency difference between the target miRNAs and RNU6-2 was less than 5%, confirming comparable amplification performance and supporting the validity of the 2^−ΔΔCt^ method.

Inter-replicate variability was assessed using the standard deviation of the Ct values. Replicates with a Ct standard deviation greater than 0.3 were re-examined, and predefined exclusion criteria were applied when necessary. The mean Ct value of the accepted replicates was used for the ΔΔCt calculation.

### 2.4. Statistical Analysis

Statistical analyses were performed using IBM SPSS Statistics for Windows (https://www.ibm.com/products/spss-statistics, accessed one 22 July 2026), Version 23.0 (IBM Corp., Armonk, NY, USA). The normality of data distribution was evaluated using the Shapiro–Wilk test. Categorical variables, such as sex distribution between groups, were compared using Yates’ continuity correction. For continuous variables, independent two-sample *t*-tests were utilized to compare normally distributed data between two groups, whereas the Mann–Whitney U test was applied for non-normally distributed data. Group comparisons of miR-221 and miR-222 relative expression levels were specifically executed on individual ΔΔCt values using Welch’s *t*-test to account for potential unequal variances. Bonferroni correction was applied to control for Type I error inflation during multiple pairwise comparisons.

Correlations between continuous parameters were evaluated using Pearson’s correlation coefficient for normally distributed data and Spearman’s rho rank correlation coefficient for non-normally distributed data. Data transformations were deliberately avoided, as rank-based non-parametric tests were deemed more robust and methodologically appropriate. Quantitative data are presented as mean ± standard deviation (SD) for normally distributed parameters, and as median (minimum–maximum) for non-normally distributed variables. Categorical data are expressed as frequencies and percentages [*n*(%)]. In addition to *p*-values, the Hodges–Lehmann median difference was calculated to estimate the clinical effect size of the differences between groups. Statistical significance was established at a two-tailed *p* < 0.050.

## 3. Results

Thirty-six patients with RRMS, seven with primary progressive MS, and thirty-seven controls were included in the study. There was no significant difference in the sex distribution between the groups (*p* = 0.400). Female patients constituted 67.40% of the patient group and 78.40% of the control group. There was no significant difference in age between the groups (*p* = 0.145). The median age of the patient group was 39.0 years, while that of the control group was 33.0 years. At the same time, the average year of MS diagnosis in the patient group was 5.05 years (minimum of one year and maximum of 25 years). The average EDSS score of the patients was 1.35, with a minimum score of 0 and a maximum score of 8. MRI involvement in 93% of the patients was cerebral, and the initial symptom of 65.10% of the patients was cortical findings (Table 1).

Independent-samples Welch’s *t*-tests were conducted to determine if there were statistically significant differences in miR-221, miR-222, and IL-23 levels between the patient (*n* = 43) and control (*n* = 37) groups. There was a statistically significant difference in miR-221 levels between the groups. Patients demonstrated significantly higher levels (M = 1.73, SD = 1.18) compared to the healthy controls (M = 1.21, SD = 0.67), t(68.0) = −2.49, *p* = 0.015, Cohen’s d = −0.55. A statistically significant difference was also observed for miR-222. The patient group exhibited significantly higher levels (M = 4.10, SD = 6.40) than the control group (M = 1.21, SD = 0.72), t(43.2) = −2.94, *p* = 0.005, Cohen’s d = −0.64. Conversely, there was no statistically significant difference in IL-23 levels between the patient group (M = 140.10, SD = 23.48) and the control group (M = 147.10, SD = 21.86), t(77.4) = 1.38, *p* = 0.171, Cohen’s d = 0.31 (Table 2).

There were no significant differences in miRNA types in MS patients according to sex, MRI involvement regions, and initial symptoms. Table 3 presents a comparison of miR-221, miR-222 and IL-23 parameters according to the gender of the patients, MRI involvement areas, and initial symptoms. Similarly, there was no statistically significant relationship between age and MS duration, EDSS, and miR-221 and miR-222 relative expression (*p* > 0.050), see Table 4.

miR-221 showed no significant correlation with IL-23 (r = −0.073; *p* = 0.644), and miR-222 likewise showed no significant correlation (r = 0.066; *p* = 0.676); both coefficients were near zero and non-significant. The subgroup analysis results indicated no statistically significant differences between the RRMS and PPMS groups (miR-221: *p* = 0.084; miR-222: *p* = 0.610; IL-23: *p* = 0.834).

## 4. Discussion

In the present study, we examined the association of miRNA-221 and miRNA-222 relative expression with EDSS scores, initial symptoms, and MRI lesion localization in newly diagnosed, naive patients with RRMS, relationships that have not been studied previously. In an experimental model performed on the murine intestine, the expression of miRNA-221/222 was induced by proinflammatory cytokines and repressed by transforming growth factor-beta (TGF-β). In that pathway, IL-23 stimulation is suppressed by negative feedback at the miRNA level, which subsequently results in the suppression of amplified inflammation. Prior work demonstrated that miRNA-221 and miRNA-222 are integral to the intestinal Th17 cell response induced after IL-23 stimulation to constrain the magnitude of the proinflammatory response [21]. In our study, miRNA-221/222 expression levels were found to be higher in MS patients compared to the control group, while IL-23 levels were not found to be different from the control group. Because the present analysis is cross-sectional and observational, and because the correlation between miRNA-222 and IL-23 was near zero and non-significant, no direct suppressive or causal effect of miRNA-221/222 on IL-23 can be inferred. Experimental data showing that miRNA-221/222 act as negative feedback regulators downstream of IL-23 represent a cellular loop that cannot be definitively confirmed by the present observational design [21].

Another study concerning miRNAs demonstrated a positive correlation between the upregulation of miRNA-21, miRNA-146a, and miRNA-146b within the cerebrospinal fluid (CSF) of MS patients exhibiting active lesions. Although serum miRNA levels may not fully capture highly compartmentalized, CNS-restricted immune processes, accumulating evidence suggests that circulating miRNAs actively reflect systemic immune activation relevant to MS pathophysiology and correlate significantly with disease-associated immune signatures [11,27].

A longitudinal study previously investigated the relationship between miRNAs and neuroimaging parameters. In that report, miRNA-22-3p, miRNA-361-5p, and miRNA-345-5p were identified as the most valid discriminators of brain atrophy and lesion load on MRI [28]. However, we did not find any statistically significant relationship between miRNA-221/222 levels and MRI findings in our cohort. This lack of correlation may be attributed to our relatively small sample size, or it might suggest the genuine absence of a biological correlation between these specific variables.

Another argument suggests that miRNA-221 may regulate the endothelial inflammatory response in vascular diseases such as atherosclerosis. It suppresses nitric oxide (NO) synthesis in human umbilical vein endothelial cells and activates the nuclear factor-kappa B (NF-κB) signaling pathway. Based on these findings, it is speculated that miRNA-221/222 might be a biomarker for atherosclerosis. It has been suggested that it could act as a similar marker in MS [29]. Interestingly, in our study, we found that miRNA-222 levels were significantly increased in patients compared to the control group.

Numerous studies suggest that miRNAs play an important role in regulating various aspects of innate immunity. These functions include the direct regulation of microbial clearance, cytokine production, and antigen presentation by MHC molecules. These innate pathways are effective in initiating antigen-specific responses, which are subsequently carried out by cells of the adaptive immune system [30].

In our study, miRNA-221/222 relative expression was not associated with lesion burden, EDSS scores, or sex. However, these levels were significantly higher in the MS group than in the control group, supporting their potential involvement in immune processes. Moreover, the observed moderate-to-large effect size for miRNA-221/222 highlights the need for further evaluation in larger cohorts to clarify their specific roles and clinical utility in distinguishing RRMS from PPMS.

The differentiation of naive CD4+ T cells into the Th17 subset is mediated by IL-21 and IL-23, with Th17 cells being generally involved in inflammation and identified by their IL-23R expression [31,32]. Comparing clinical studies on the relationship between IL-23 and MS remains challenging due to the heterogeneity and inclusion of different patient populations. Nevertheless, anti-IL-23 treatment studies have shown that targeting this pathway inhibits multiple inflammatory signaling pathways and prevents disease relapse, which is achieved by a reduced expression of IFN-γ, IL-10, IL-17, IL-6, and TNF in the CNS, as well as decreased serum levels of IL-17 [33]. Clinically, serum IL-23 levels in MS patients appear to fluctuate based on disease activity; for instance, a study involving 30 RRMS patients and 30 controls reported significantly elevated IL-23 levels during the acute attack period, whereas this difference completely disappeared one month after the attack [34]. In contrast, a study comparing 40 RRMS patients in the remission period with controls—28 of whom were using interferon-beta—found that IL-23 levels in MS patients were significantly higher without any relation to interferon use [18]. Adding to this complexity, another study reported no significant difference in Th17-related cytokines (IL-17, IL-23, and IL-26) between controls and MS patients before starting treatment, but documented that IL-23 and IL-26 levels increased gradually with IFN-β1 treatment [35]. In light of the aforementioned literature and our findings, serum IL-23 levels appear to fluctuate depending on disease clinical phases, such as acute attacks or remissions, as well as therapeutic interventions. Accordingly, our finding of comparable serum IL-23 concentrations between the study groups should not be interpreted as definitive evidence that IL-23 signaling is unaltered in MS pathophysiology. Several confounding factors must be considered: first, all patients were sampled during the remission phase, a period when circulating IL-23 concentrations may reach their lowest baseline; second, standard serum ELISA methodology may lack the analytical sensitivity required to detect compartmentalized or low-abundance cytokine variations; third, IL-23 is known to exhibit inherent circadian and assay-dependent variability; and finally, peripheral serum levels do not necessarily mirror localized central nervous system or cerebrospinal fluid immune activity. Consequently, in the absence of concurrent cerebrospinal fluid measurements and longitudinal, activity-stratified sampling, this null serum IL-23 result remains inconclusive rather than indicative of normal underlying IL-23 biology. The miRNA-221 inhibitor LNA-i-miRNA-221 provides a new investigational clinical option. Preliminary phase I study results are of great interest in moving toward further phases of clinical development to better define and characterize the effectiveness and safety profile of this agent in specific neoplasms. A major novelty will be to consider LNA-i-miRNA-221 in chemoprevention and anti-inflammatory strategies, thereby opening a novel research avenue on the translational relevance of these therapeutic targets against both malignant and non-malignant diseases [36].

Our study had several limitations that warrant consideration. First, the sample size was relatively modest (*n* = 43 patients), leaving the subgroup comparisons—stratified by sex, MRI region, initial symptoms, and MS subtypes, as well as correlations with age, EDSS scores, and disease duration—underpowered despite the application of Bonferroni correction. Consequently, these statistical analyses, including the RRMS-versus-PPMS contrast (where seven PPMS patients were enrolled as recruitment allowed), should be regarded strictly as exploratory and hypothesis-generating; their null findings do not constitute proof of equivalence. Second, the cross-sectional and serum-based study design preclued any causal or temporal inferences, potentially missing localized, CNS-compartmentalized immunobiological processes. Furthermore, all patients were sampled during clinical remission, which—coupled with the assay-sensitivity and circadian variability considerations noted above—limits the definitive interpretation of the serum IL-23 findings. Third, because the control group served as the calibrator in the 2^−ΔΔCt^ method, between-group statistical comparisons were appropriately executed on individual ΔΔCt values. To resolve these open questions, broader prospective studies spanning all MS phenotypes (including radiologically isolated syndrome) are needed. Ideally, such future research should incorporate paired serum and CSF sampling, activity-stratified longitudinal measurements, and an adult-versus-pediatric cohort comparison.

## 5. Conclusions

In conclusion, serum miRNA-221 and miRNA-222 relative expression levels were significantly higher in treatment-naive MS patients than in matched controls, whereas serum IL-23 concentrations did not differ between the groups, and no significant subgroup associations were detected. These clinical data demonstrate a systemic association rather than establishing a pathogenic or regulatory role. Nevertheless, on the basis of prior experimental literature, our findings suggest that miRNA-221/222 could participate in IL-23–Th17 pathway regulation and might serve as accessible circulating biomarkers. Any definitive causal, mechanistic, CNS-compartmentalized, or longitudinal/activity-related roles remain unknown at present; resolving these questions will require functional assays, cerebrospinal fluid sampling, and larger prospective study designs.

## Figures and Tables

**Table 1:** Demographic and clinical characteristics.

Variable	MS Patients (*n* = 43)	Controls (*n* = 37)	Total (*n* = 80)	Test/*p*
Female sex	29 (67.4%)	29 (78.4%)	58 (72.5%)	Yates chi-square = 0.708; *p* = 0.400
Male sex	14 (32.6%)	8 (21.6%)	22 (27.5%)	
Age, years	39.47 ± 10.75; 39.00 (20–60)	36.19 ± 9.38; 33.00 (23–54)	37.95 ± 10.21; 37.00 (20–60)	Mann–Whitney U = 644.500; *p* = 0.145
MS diagnosis duration, years	5.05 ± 5.90; 2.00 (1–25)	—	5.05 ± 5.90; 2.00 (1–25)	—
EDSS	1.35 ± 2.23; 0.00 (0–8)	—	1.35 ± 2.23; 0.00 (0–8)	—
MRI involvement: optic	4 (9.3%)	—	4 (9.3%)	—
MRI involvement: cerebral	40 (93.0%)	—	40 (93.0%)	—
MRI involvement: brainstem	12 (27.9%)	—	12 (27.9%)	—
MRI involvement: spinal	19 (44.2%)	—	19 (44.2%)	—
MRI involvement: cerebellar	8 (18.6%)	—	8 (18.6%)	—
Initial symptom: optic involvement	8 (18.6%)	—	8 (18.6%)	—
Initial symptom: spinal involvement	19 (44.2%)	—	19 (44.2%)	—
Initial symptom: cortical findings	28 (65.1%)	—	28 (65.1%)	—

Values are presented as frequency (%), mean ± standard deviation, and median (minimum–maximum). EDSS: Expanded Disability Status Scale; MRI: magnetic resonance imaging.

**Table 2:** Comparison of miR-221, miR-222, and IL-23 between patients and controls.

Variable	Patients (*n* = 43)	Controls (*n* = 37)	t	*p*	Cohen’s d
miR-221 (fold change)	1.73 ± 1.18 1.32 (0.12–4.67)	1.21 ± 0.67 1.14 (0.17–3.37)	−2.49	0.015 *	−0.55
miR-222 (fold change)	4.10 ± 6.40 1.74 (0.23–29.65)	1.21 ± 0.72 1.14 (0.20–3.56)	−2.94	0.005 **	−0.64
IL-23 (pg/mL)	140.10 ± 23.48 137.90 (94.73–180.00)	147.10 ± 21.86 150.00 (88.30–180.00)	1.38	0.171	0.31

Data are presented as mean ± standard deviation and median (minimum–maximum). SD, standard deviation; d, Cohen’s d effect size. The t and *p* values were obtained from the independent two-sample *t*-test. * *p* < 0.05; ** *p* < 0.01.

**Table 3:** Comparison of miR-221, miR-222 and IL-23 parameters according to the gender of the patients, MRI involvement areas, and initial symptoms.

			miRNA-221	miRNA-222	IL-23
Sex					
	Female		1.82 ± 0.23 1.36 (0.12–4.67)	1.84 ± 1.12 1.65 (0.23–4.56)	141.17 ± 4.44 142.33 (94.72–180)
	Male		1.55 ± 0.33 1.16 (0.39–4.51)	4.01 ± 1.08 1.75 (0.23–23.42)	137.98 ± 6.21 137.67 (95.87–180)
		Test statistics	179	181.5	186.5
		*p*	0.534 *	0.577 *	0.669
MRI involvement					
	Optic involvement		2.43 ± 1.61 2.55 (0.48–4.15)	6.02 ± 7.26 3.07 (1.25–16.68)	148.44 ± 11.15 148.13 (136.39–161.12)
	Cortical		1.72 ± 1.19 1.27 (0.12–4.67)	4.10 ± 6.40 1.69 (0.23–29.65)	140.23 ± 24.35 139.33 (94.73–180)
	Brainstem		1.42 ± 1.09 1.18 (0.39–4.51)	4.56 ± 9.02 1.35 (0.23–29.65)	138.21 ± 17.68 137.93 (95.87–161.85)
	Spinal		1.79 ± 1.29 1.32 (0.39–4.67)	4.27 ± 6.81 1.57 (0.23–23.43)	140.13 ± 23.47 149.61 (94.7–180)
	Cerebellar		1.31 ± 0.85 1.15 (0.39–2.84)	7.10 ± 11.75 1.61 (0.23–29.65)	141.38 ± 13.75 137.20 (119.22–161.85)
		*p* **	˃0.050	˃0.050	˃0.050
Initial symptom					
	Optic involvement		2.08 ± 1.39 1.60 (0.39–4.15)	4.91 ± 6.15 1.36 (0.45–16.68)	142.98 ± 21.64 139.36 (116.31–180)
	Spinal involvement		1.90 ± 1.22 1.68 (0.39–4.67)	5.51 ± 8.39 2.51 (0.31–29.65)	140.24 ± 26.76 137.73 (94.7–180)
	Cortical findings		1.70 ± 1.19 1.25 (0.12–4.67)	4.00 ± 6.71 2.15 (0.23–9.45)	136.06 ± 22.63 136.95 (94.7–180)
		*p* **	˃0.050	˃0.050	˃0.050

* Mann–Whitney U test; ** Bonferroni correction, mean ± s.d, median (min–max).

**Table 4:** Examination of the relationship between patients’ age, year of MS diagnosis, and EDSS.

	Age		MS Diagnosis Year		EDSS	
	r	*p*	r	*p*	r	*p*
miRNA-221	−0.110	0.483	0.271	0.172	0.120	0.445
miRNA-222	−0.135	0.388	−0.187	0.23	−0.058	0.713
IL-23	−0.015 *	0.922	0.130 **	0.406	0.011 **	0.944

* Pearson correlation coefficient, ** Spearman’s correlation coefficient.

## Data Availability

De-identified clinical data, IL-23 measurements, raw Ct data, ΔCt values, relative-expression calculations, and available pre-analytical sampling information are available from the corresponding author upon reasonable request, subject to institutional and ethics committee requirements.

## Abbreviations

BMI	Body Mass Index
cDNA	Complementary DNA
CNS	Central Nervous System
CSF	Cerebrospinal Fluid
Ct	Cycle Threshold
DMT	Disease-Modifying Therapy
EDSS	Expanded Disability Status Scale
ELISA	Enzyme-Linked Immunosorbent Assay
IL	Interleukin
JAK	Janus Kinase
miRNA	microRNA
MRI	Magnetic Resonance Imaging
MS	Multiple Sclerosis
PCR	Polymerase Chain Reaction
PPMS	Primary Progressive Multiple Sclerosis
RT-qPCR	quantitative reverse-transcription polymerase chain reaction
RNU6-2	RNA, U6 small nuclear 2
ΔΔCt	Delta–Delta Cycle Threshold
RORγt	Retinoid-Related Orphan Receptor-γt
RRMS	Relapsing–Remitting Multiple Sclerosis
SPMS	Secondary Progressive Multiple Sclerosis
STAT	Signal Transducer and Activator of Transcription
TGF-β	Transforming Growth Factor Beta
Th	T helper
TNF	Tumor Necrosis Factor
Tyk	Tyrosine Kinase
